# Psychometric evaluation of age- and culture-appropriate Hindi trauma-related questionnaires for children and adolescents

**DOI:** 10.1186/s13034-025-01018-9

**Published:** 2025-12-30

**Authors:** Lora Stier, Jan H. Kamphuis, Vipasha Goyal, Nitya Shah, Arnold A. P. van Emmerik

**Affiliations:** Department of Psychology, University of Amsterdam, Nieuwe Achtergracht 129-B, Amsterdam, 1018 WS The Netherlands

**Keywords:** Trauma, Children, Adolescents, India, Hindi, Psychometric validation, CRIES-13, CPTCI, DSRS-C

## Abstract

**Background:**

Given India’s high rates of trauma exposure and mental health service shortages, age- and culture-appropriate self-report tools may enhance detection and treatment of trauma-related symptoms in low-resource settings. This study psychometrically evaluated Hindi versions of three trauma-related questionnaires: the Children’s Revised Impact of Event Scale (CRIES-13), the Child Post-Traumatic Cognitions Inventory (CPTCI), and the Depression Self-Rating Scale for Children (DSRS-C), that were adapted for children and adolescents in previous research.

**Methods:**

A total of 305 Hindi-speaking participants aged 6–18 completed the questionnaires online. Confirmatory factor analyses and Cronbach’s alpha were conducted to evaluate the internal structure and internal consistency of the questionnaires, and Pearson’s correlations were computed to evaluate their convergent validity.

**Results:**

The CRIES-13 best fits a three-factor model, the CPTCI a two-factor model, and the DSRS-C a two-factor model. Internal consistency was acceptable to excellent across scales, except for the Arousal subscale of the CRIES-13. Convergent validity was supported by moderate to strong intercorrelations and associations with trauma exposure indices.

**Conclusions:**

The adapted Hindi instruments are psychometrically promising tools for assessing trauma-related symptoms among Indian youth that could inform the diagnosis and treatment of trauma-exposed populations. Limitations and future research directions are discussed.

## Introduction

Approximately 20% and 12% of children and adolescents exposed to trauma develop Posttraumatic Stress Disorder (PTSD) under the DSM-IV and DSM-5 criteria, respectively, according to a meta-analysis of predominantly North American and European samples [1]. In addition to symptoms such as intrusions, persistent avoidance, negative alterations in cognitions and mood, and changes in arousal and reactivity associated with this disorder [2], children who have experienced trauma often struggle with managing their emotions [3] and face disruptions in cognitive control processes, which can affect attention, working memory, and impulse control [4]. Despite these far-reaching effects of trauma, PTSD in children and adolescents likely remains considerably underdiagnosed, particularly in emerging countries where clinical resources are limited [5].

One such country is India, which has the world’s second largest population and is home to one of the largest child and adolescent populations, exceeding 450 million [6]. Furthermore, India’s vast population suffers from high trauma exposure. Specifically, natural disasters such as floods, droughts, and earthquakes affect 30 million people and cause approximately 4,000 deaths annually [7]. A case in point, the 2004 tsunami alone claimed over 10,200 Indian lives, displaced 112,500 people, and left more than 5,800 missing [8]. India also has the highest rate of road fatalities worldwide, accounting for 11% of global traffic deaths, with 1,5 million deaths each year, making road accidents the leading cause of death among youth aged 5 to 29 [9, 10]. Exposure to interpersonal violence is also common among children, with nearly 53% reporting abuse by a parent, relative, or teacher [11]. Children in slum areas, where 17.4% of urban households live, face heightened vulnerability due to unstable living conditions [12]. Taken together, the Indian population experiences high trauma exposure, likely resulting in a significant mental health burden, although the lack of standardized measures makes it challenging to make reliable prevalence estimates. Compounding this issue is a severe shortage of mental health professionals, particularly in rural areas, along with inadequate infrastructure, widespread stigma, and poor integration of mental health services into primary healthcare, which further widens the treatment gap [13].

Self-report questionnaires for trauma-related problems may help to address this problem, as they can be relatively easily distributed and administered in resource-limited settings [14]. However, cultural factors can greatly influence how symptoms are expressed, interpreted, and reported [15]. In India, for example, psychological distress often manifests as somatic complaints, such as headaches or fatigue, rather than as emotional or cognitive issues, as is common in Western contexts [16]. If these cultural differences are ignored, healthcare providers may misinterpret such physical symptoms as purely somatic problems, potentially overlooking their psychological origins. This can lead to misguided diagnosis and treatment, negatively impacting clinical outcomes [17]. Therefore, cultural adaptation of assessment tools is essential to effective identification and subsequent treatment of mental health issues.

India has two official languages, Hindi and English. Hindi is the most widely spoken, with around 44% of the population, i.e., over 528 million people, having it as their first language [18]. While English is also an official language, its use is limited to governmental, legal, and business contexts. The present study therefore evaluated Hindi versions of three questionnaires commonly used in trauma research and practice in children and adolescents: the Children’s Revised Impact of Event Scale (CRIES-13) [19], the Child Post-Traumatic Cognitions Inventory (CPTCI) [20], and the Depression Self-Rating Scale for Children (DSRS-C) [21, 22]. These questionnaires have shown favorable psychometric properties for screening children and adolescents for posttraumatic stress symptoms (CRIES-13), trauma-related cognitions (CPTCI), and comorbid depressive symptoms (DSRS-C). More specifically, robust evidence supports the reliability and internal consistency of the measures in pertinent samples [23–25]. Previous research has also provided robust evidence for aspects of construct validity, convergent validity, discriminant validity, and criterion validity [26–28]. Studies of the internal structure of these questionnaires have yielded somewhat inconsistent findings, however. While some studies propose single-factor structures for all three questionnaires [29–31], others suggest two-factor structures for all three questionnaires [20, 23, 32, 33] or a three-factor structure for the CRIES-13 [34, 35]. Therefore, we tested all proposed models to identify the best-fitting factor structure of our Hindi versions of these questionnaires.

In addition to charting the trauma history of our sample, the present study aimed to [1] determine the factor structure, and [2] evaluate the internal consistency and convergent validity of three trauma-related questionnaires (CRIES-13, CPTCI, DSRS-C) among children and adolescents in India. The original English versions of these questionnaires were previously translated into Hindi for use in an efficacy study of eye movement desensitization and reprocessing (EMDR) treatment in former child slaves in India [36]. The translation followed a formal forward (into Hindi) and back-translation (into English) process, after which discrepancies between the original and back-translated English versions were resolved through discussion. Based on our experiences in that study, several adaptations to these initial translations were made, including correcting some grammatical errors and inaccurate translations, as well as simplifying some phrasings to make the questionnaires more child friendly. Regarding our first aim, we expected the factor structures to align with the most recent high-quality studies that we could identify. Specifically, we expected the CRIES-13 to exhibit a three-factor structure (35), CPTCI a two-factor structure (23), and DSRS-C a two-factor structure (32). Regarding our second aim, we expected the questionnaires and their subscales to have at least acceptable internal consistency (Cronbach’s alpha ≥ 0.70) and good convergent validity as evidenced by at least moderate (*r* ≥ .30, *p* < .05) intercorrelations and correlations with trauma exposure indices and by higher scores for interpersonal compared to non-interpersonal trauma.

## Methods

### Participants

Participants were recruited in India through various online platforms and a network of teachers, psychologists, researchers, and acquaintances with access to Indian children and adolescents. To be included in our study, children and adolescents (a) were required to be between 6 and 18 years old and (b) demonstrate age-appropriate literacy in Hindi.

To determine our requisite sample size, we followed recommendations from previous research on confirmatory factor analysis (CFA). Clark and Watson [37] suggest a minimum of 200 to 300 participants, while Guadagnoli and Velicer [38] recommend a minimum sample size of 300 participants to achieve stable and replicable results for factor solutions with few variables per component and low component loadings, and Comrey [39] suggests a minimum of 200 participants for factor analysis with 40 or fewer variables. In line with these recommendations, we aimed at a sample size of 300 participants to ensure robust findings.

Of 2841 participants who began our online survey, we retained only responses with a progress value of 100% to ensure that participants had viewed all survey items. This reduced the dataset from 2,841 to 565 cases. Subsequently, 66 responses automatically identified by Qualtrics as spam—typically indicative of automated or fabricated entries—were removed, along with four test submissions generated by the research team, resulting in 495 valid records. Of these, 145 lacked parental consent and 36 lacked self-consent, yielding 314 eligible cases. Finally, nine respondents reported being over 18 years of age, resulting in a final analytic sample of 305 participants. Their age ranged between 6 and 18 (*M* = 13.07; *SD* = 2.53) and 116 (38.0%) identified as female, 188 (61.6%) as male, and one (0.3%) as non-binary. Most of the sample resided in Bhopal (Madhya Pradesh; *n* = 125, 41.0%) and Bilaspur (Chhattisgarh; *n* = 127, 41.6%), although children and adolescents from other regions of India also participated (details available from the authors upon reasonable request). Most participants identified as adhering to Hinduism (*n* = 247; 81.0%), followed by Islam (*n* = 27; 8.9%), Christianity (*n* = 22; 7.2%), Other religions (*n* = 6; 2.0%), and Buddhism (*n* = 3; 0.9%).

### Instruments

### Children’sRevised Impact of Event Scale

The Children’s Revised Impact of Event Scale (CRIES-13) [19] is a widely used tool for screening trauma-related symptoms in children aged between 8 and 18. It has been translated into more than 25 languages [34, 36, 40]. The CRIES-13 has three subscales that measure intrusion, avoidance, and arousal symptoms and includes 13 items that are rated on a 4-point scale ranging from Not at all = 0, Rarely = 1, Sometimes = 3 to Often = 5. It has shown good internal consistency and test-retest reliability [28, 34, 40] in trauma-exposed samples. A total score of 30 or higher on the CRIES-13 indicates a high likelihood of a PTSD diagnosis [30].

#### Child Post-Traumatic Cognitions Inventory

The Child Post-Traumatic Cognitions Inventory (CPTCI) [20] is a questionnaire for negative trauma-related thoughts in children and adolescents aged between 6 and 18. It consists of 25 items that are rated on a 4-point scale ranging from Strongly disagree = 1, Slightly disagree = 2, Slightly agree = 3 to Strongly agree = 4. The CPTCI has two subscales labelled “Permanent and Disturbing Change” (CPTCI-PC; 13 items) and “Fragile Person in a Scary World” (CPTCI-SW, 12 items). The total scale showed high internal consistency in children and adolescents with a history of different traumatic events [23]. Scores of 48 or higher are considered clinically significant and are often observed in children and adolescents with PTSD [41].

#### Depression Self-Rating Scale for Children

The Birleson Depression Self-Rating Scale for Children (DSRS-C) [21, 22] measures depressive symptoms in children and adolescents aged between 8 and 14. It includes 18 items that are rated on a 3-point scale ranging from Never = 0, Sometimes = 1 to Mostly = 2. The DSRS-C showed good test-retest reliability and internal consistency in depressed and non-depressed children [21, 22]. Scores of 15 or higher indicate an elevated risk of major depressive disorder or dysthymia [22].

#### Demographic characteristics and trauma history

Participants’ demographic characteristics and trauma history were collected using a questionnaire that was specifically designed for this study. In addition to questions about their gender, age, religious affiliation, and family structure, participants were presented with a list of traumatic events that was based on the Life Events Checklist for DSM-5 (LEC-5) [42]. We added two events specific to the Indian context (i.e., caste-related violence and religious riots), resulting in a list of 13 events in total. We also included an open-ended item instructing participants to report the most stressful experience in their life if they had not endorsed any of the predefined events on the list. Participants were asked to indicate if they had experienced, witnessed, or learned about these events happening to a relative or close friend, and whether the most recent event had occurred last week, in the last two weeks, last month, a few months ago, last year, or a few years ago. They were then instructed to complete the CRIES-13 and CPTCI based on the “stressful event (or events)” that they had endorsed.

### Procedure

Adaptations to the initial Hindi translations of the questionnaires were made with the help of two Research Master Psychology graduate students (VG and NS) of Indian nationality majoring in psychology at the University of Amsterdam, who had Hindi as their native and second language, respectively. In several iterative rounds, they first independently reviewed the questionnaires and proposed revisions, which were then discussed and agreed upon in conjunction with the first author (LC). This resulted in minor adaptations of the initial translations [36] (see Appendix A-C for the adapted and original versions of the questionnaires). Next, an online survey was created and distributed through a link or QR code. The survey successively included an information letter and consent forms for parents or legal guardians (if children were under 16 years old) and for the children themselves, the questionnaires described above, and an evaluation of the participants’ self-reported comprehension of the survey on a scale ranging from 0 (“I did not understand any of the questions I was asked”) to 100 (“I understood every question I was asked”). The average conprehension rating was 81.77 (*SD* = 24.62) in the final sample (*N* = 305), indicating generally good self-reported understanding of the survey content. The study procedures were approved by the Ethics Review Board of the Faculty of Social and Behavioural Sciences at the University of Amsterdam (ID: FMG-2908).

### Data analysis

All statistical analyses were conducted using R software [43] and relevant packages. We stratified participants based on the age range of the original target population of each questionnaire (i.e., 8–18 for the CRIES-13, 6 to 18 for the CPTCI, and 8–14 for the DSRS-C).

The Lavaan package [44] was used to perform Confirmatory Factor Analysis (CFA). As the scores demonstrated a non-normal distribution, we used maximum-likelihood estimation with Robust Maximum Likelihood (MLR) for model identification. We evaluated model fit using the Standardized Root Mean Square Residual (SRMR), Root Mean Square Error of Approximation (RMSEA), Comparative Fit Index (CFI), and Tucker-Lewis Index (TLI), with recommended thresholds of CFI and TLI > 0.90 and RMSEA and SRMR < 0.08 for a good fit [45, 46]. Lower values of the Akaike Information Criterion (AIC) and Bayesian Information Criterion (BIC) indicated better fit and were used for model comparison. Modification Indices (MIs) were used for assessing misfit, with MIs exceeding 3.84 suggesting potential model revisions. Factor loadings were deemed statistically meaningful if ≥ 0.5 and *p* < .05. Factor correlations needed to be < 0.85 to indicate distinct factors.

The internal consistency of the questionnaires was assessed using Cronbach’s alpha for the total scales as well as comprising subscales. Convergent validity was assessed by computing Pearson’s correlations between the CRIES-13, CPTCI, DSRS-C, and trauma history scores. Trauma history scores were calculated as the total number of events that a child had experienced directly, observed, or learned about. Convergent validity was further explored by evaluating the relationship between these types of exposure and the severity of trauma-related symptoms. A trauma severity index was developed by combining the number of traumatic events with type of exposure (Directly experienced = 3, Observed = 2, Learned about = 1) and examining if this index exhibited positive Pearson’s correlations with the CRIES-13, CPTCI, and DSRS-C scores. Additionally, we investigated whether symptom severity scores were significantly higher for interpersonal trauma compared to non-interpersonal trauma using Welch’s *t*-tests (due to unequal variances), and whether these scores showed negative Pearson’s correlations with time elapsed since the most recent traumatic event. To control for Type I errors resulting from multiple testing, a Bonferroni correction was applied, with a significance level set at *p* < .01.

## Results

### Descriptives

**Fig. 1:**
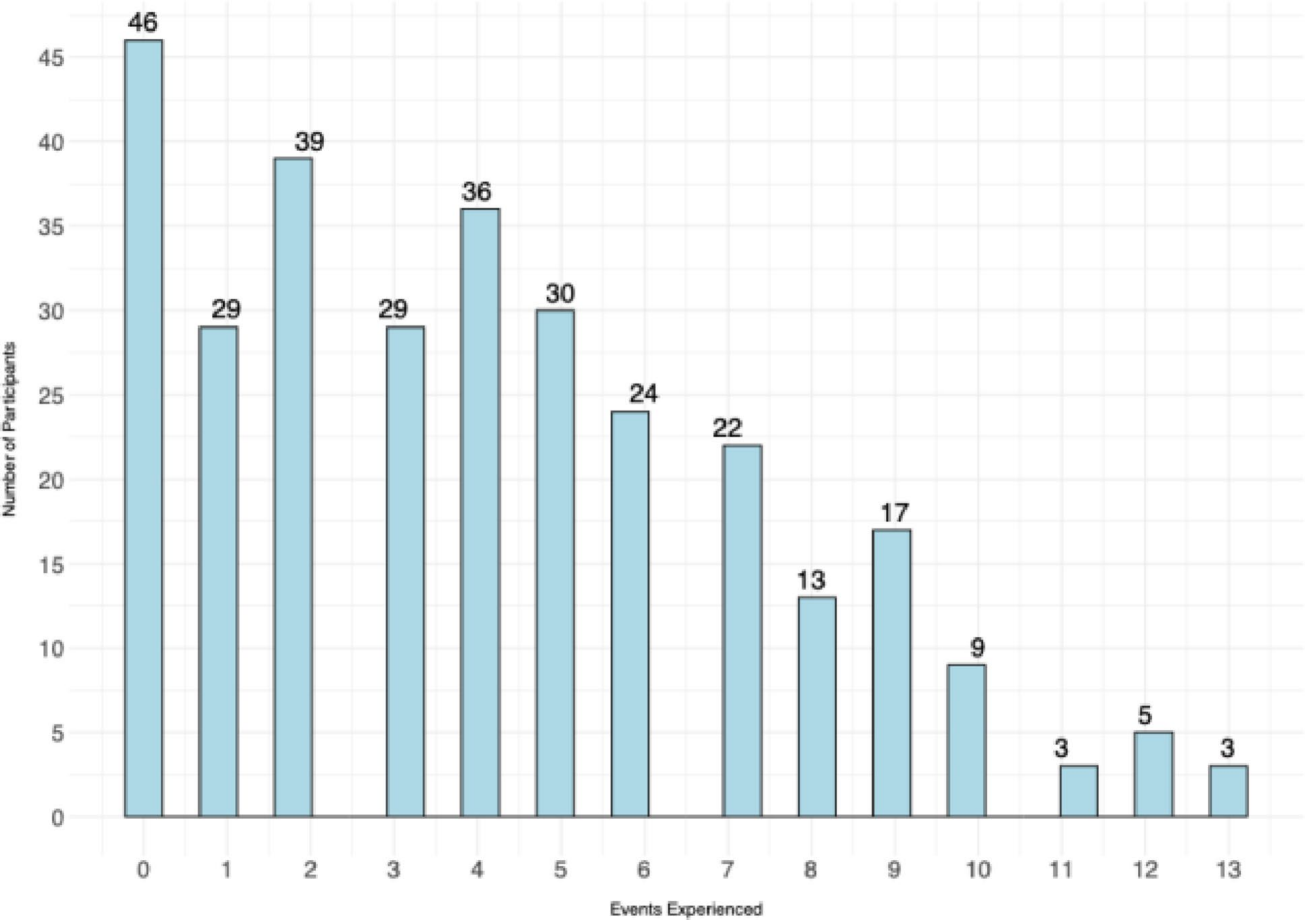
Distribution of the Number of Experienced Events

Participants reported having experienced an average of 4 traumatic events (*M* = 4.15, *SD* = 3.23, range 0–13). Table 1 details the trauma history of our participants, and Fig. 1 shows the distribution of the number of self-reported traumatic events on the 13 predefined LEC-5 items regardless of whether these events were directly experienced, witnessed, or learned about. Descriptive statistics for the CRIES-13, CPTCI, and DSRS-C are presented in the Table 2.

**Table 1:** *Types of traumatic events and exposure modes of participants (*N *= 305)*

Traumatic event	Directly experienced	Witnessed	Learned about
Natural disaster	17	40	28
Transportation accident	24	67	50
Injury	142	34	17
Physical assault	24	41	17
Assault with a weapon	6	18	17
Sexual assault	11	17	12
Combat or war exposure	4	20	6
Serious illness	35	49	76
Serious injury	43	49	52
Death	0	100	66
Divorce	2	10	22
Caste discrimination	12	64	13
Religious riots	3	49	8

**Table 2:** Descriptive statistics for Trauma-Related questionnaires

Questionnaire	*n*	M	SD	Range (possible)	% above cutoff
CRIES-13	298	16.33	12.65	0–61 (0-65)	13.8%
CPTCI	305	42.55	15.40	25–100 (25-100)	31.1%
DSRS-C	208	7.50	5.78	0–30 (0-36)	13.0%

Note. CRIES-13 = Children’s Revised Impact of Events Scale; CPTCI = Child Post-Traumatic Cognitions Inventory; DSRS-C = Depression Self-Rating Scale for Children. Cutoffs on the CRIES-13, CPTCI, and DSRS-C were 30, 48, and 15 or higher, respectively

### Internal structure

#### Confirmatory factor analysis

**Fig. 2:**
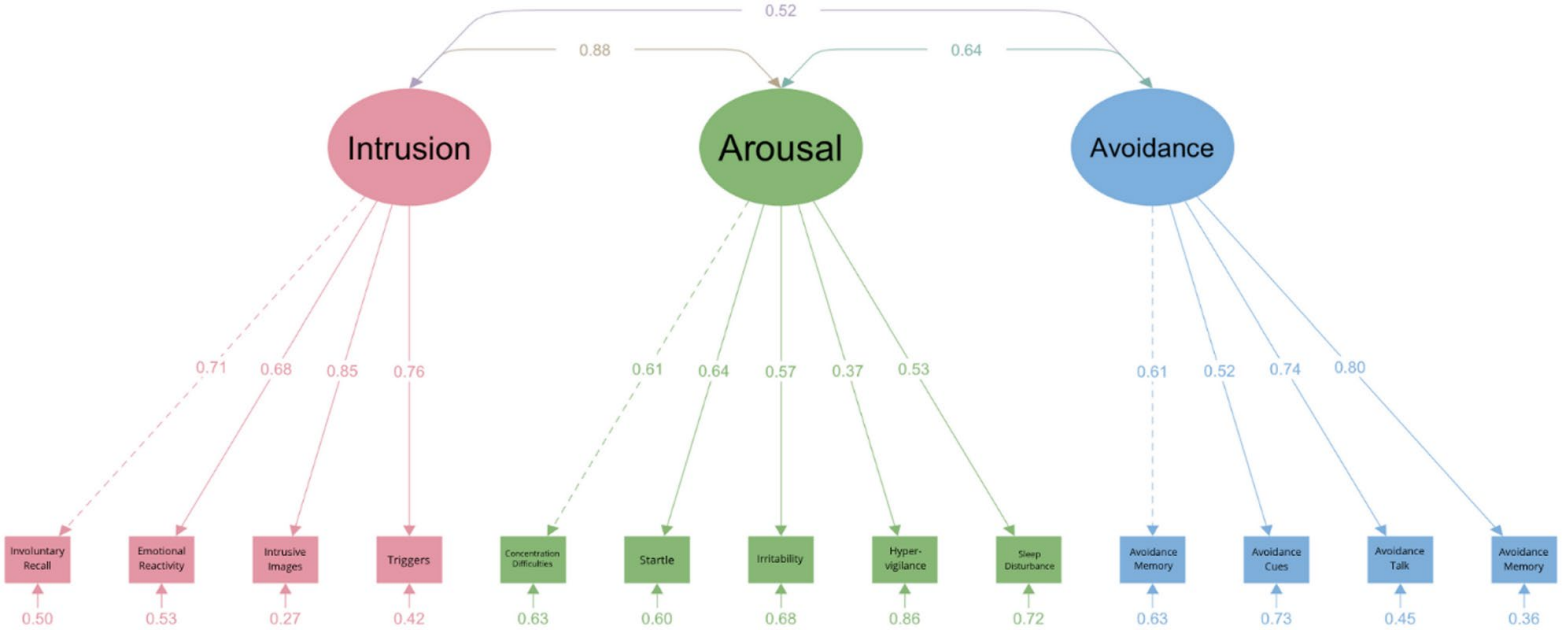
Path Diagram of the Three-Factor Model of the CRIES-13

*CRIES-13.* For the CRIES-13, we examined three models including a mono-factorial structure with three subscales (Intrusion, Arousal, and Avoidance) loading onto a higher-order PTSD factor [30], a two-factor structure with Intrusion and Arousal as one factor and Avoidance as the second factor [40], and a three-factor structure with separate factors for Intrusion, Arousal, and Avoidance [35]. The CRIES-13 scores exhibited a non-normal distribution (*W* = 0.935, *p* < .001), which led us to use the MLR estimation method because of its suitability for non-normal data. Using the Lavaan package, we evaluated three models of the CRIES-13, all of which showed significant chi-square (χ2) values (Table 3), indicating some degree of model misfit. The additional fit-indices suggested that the three-factor model (with separate factors for Arousal, Intrusion, and Avoidance; Fig. 2) showed the best fit with the CRIES-13 data. This is supported by favorable CFI and TLI values that exceed the recommended threshold of 0.90. The SRMR and RMSEA also demonstrated satisfactory fit. Furthermore, the three-factor model outperformed the other models based on lower AIC and BIC values. The factor loadings for all items were above 0.5, except for item 12 (“Are you alert and watchful even when there is no obvious need to be?“), which showed a factor loading of 0.37 on the Arousal latent variable (Fig. 2). The correlations between the latent variables were below 0.85, except for a correlation of 0.88 between the Intrusion and Arousal latent variables.

**Table 3:** Fit indices of the CRIES-13 models

*n* = 298	χ^2^ (df)	SRMR	RMSEA	90% CI	CFI	TLI	AIC	BIC	
Cut-off values		≤ 0.08	≤ 0.08	≤ 0.08	≥ 0.90	≥ 0.90			
3-factor model	169.97 (62)	0.060	0.057	0.045, 0.070	0.932	0.914	13393.27	13500.49	
2-factor model	192.65 (64)	0.066	0.062	0.050, 0.075	0.916	0.897	13411.96	13511.78	
1-factor model	351.38 (65)	0.086	0.098	0.088, 0.109	0.789	0.747	13568.68	13664.80	
Revised model	155.086 (61)	0.049	0.053	0.040, 0.066	0.941	0.925	13380.39	13491.30	

Note. CRIES-13 = Children’s Revised Impact of Events Scale

**Fig. 3:**
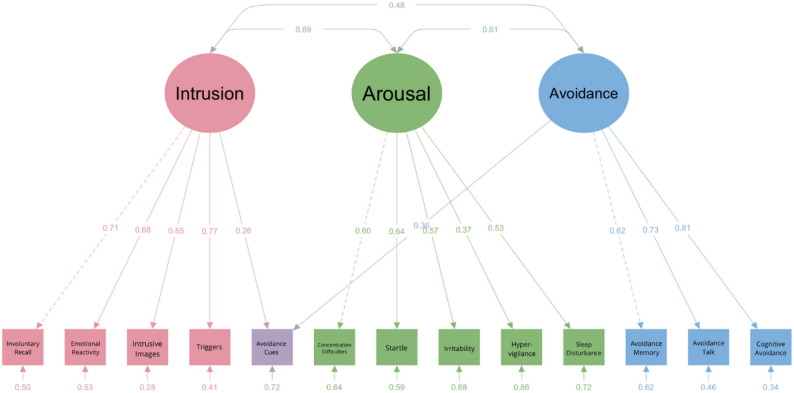
Path Diagram of the Revised Three-Factor Model of the CRIES-13

We explored potential model revisions using MIs from the misfit analysis and prioritizing modifications that balanced statistical improvement with theoretical coherence. Residual covariance between the Intrusion latent variable and item 6 (“Do you stay away from reminders of it?”) was selected due to its high MI (14.81), which suggested a notable improvement in model fit when this covariance was freed (see Table 3; Fig. 3). Consistent with theoretical expectations, this may be explained by the fact that intrusion symptoms are known to drive avoidance behaviors [47] and the item clearly inquires about avoidance behaviors. We continued our analyses with item 6 as part of the Avoidance subscale, corresponding to the subscale structure of the original CRIES-13 and in line with the revised model.

**Fig. 4:**
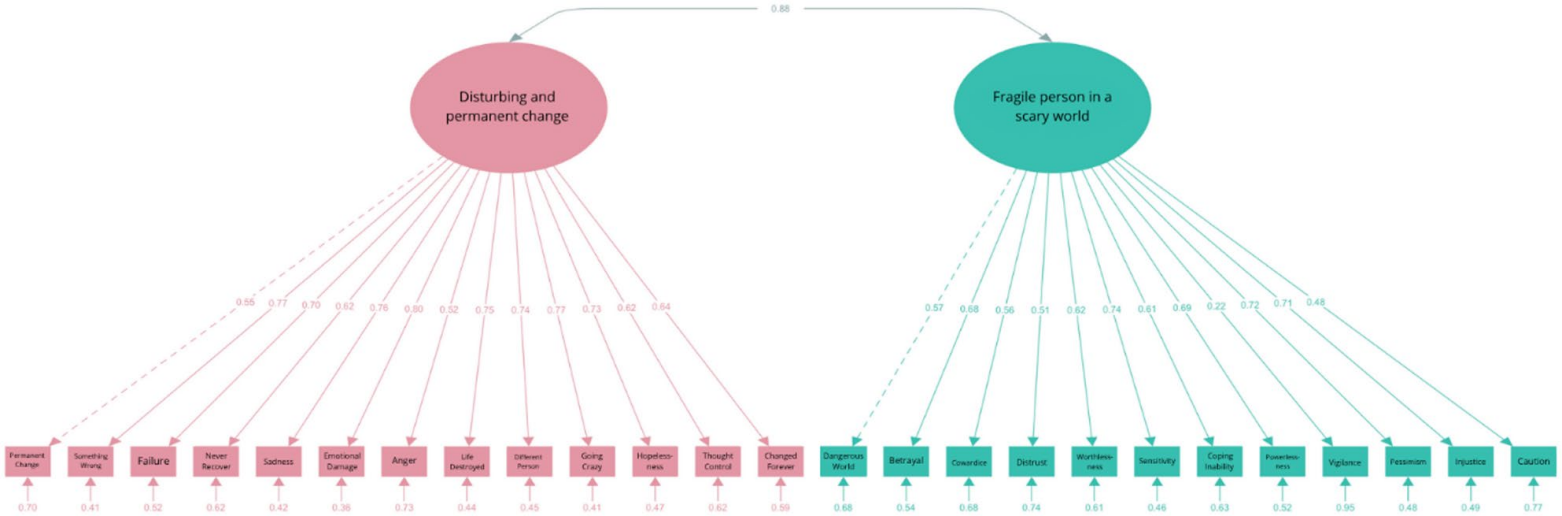
Path Diagram of the Two-Factor Model of the CPTCI

*CPTCI.* For the CPTCI, we tested two models including a mono-factorial structure (31) and a two-factor structure with CPTCI-PC and CPTCI-SW as separate factors [23]. The data from the CPTCI questionnaire exhibited a non-normal distribution (*W* = 0.902, *p* < .001), which required the use of the MLR estimation method. Confirmatory factor analysis was performed to evaluate a one-factor model and a two-factor model. Both models produced statistically significant chi-square values (χ2) at *p* < .001, again indicating some degree of model misfit. Specifically, the two-factor model achieved higher values for the CFI and TLI, and lower values for the AIC and BIC, compared to the one-factor model (see Table 4). Most factor loadings exceeded 0.5, except for item 12 (“I have to watch out for danger all the time.“) with a factor loading of 0.22 on the Fragile Person in the Scary World latent variable. Correlations between the two latent variables were strong, with a value of 0.89 (see Fig. 4).

**Table 4:** Fit indices of the CPTCI models

*n* = 305	χ^2^ (df)	SRMR	RMSEA	90% CI	CFI	TLI	AIC	BIC
Cut-off values		≤ 0.08	≤ 0.08	≤ 0.08	≥ 0.90	≥ 0.90		
2-factor model	795.91 (274)	0.058	0.057	0.052, 0.063	0.888	0.878	17764.89	17954.63
1-factor model	897.78 (275)	0.061	0.064	0.058, 0.069	0.862	0.849	17864.77	18050.78
Revised model	718.074 (272)	0.055	0.052	0.046, 0.058	0.908	0.899	17691.06	17888.24

Note. CPTCI = Child Post-Traumatic Cognitions Inventory

**Fig. 5:**
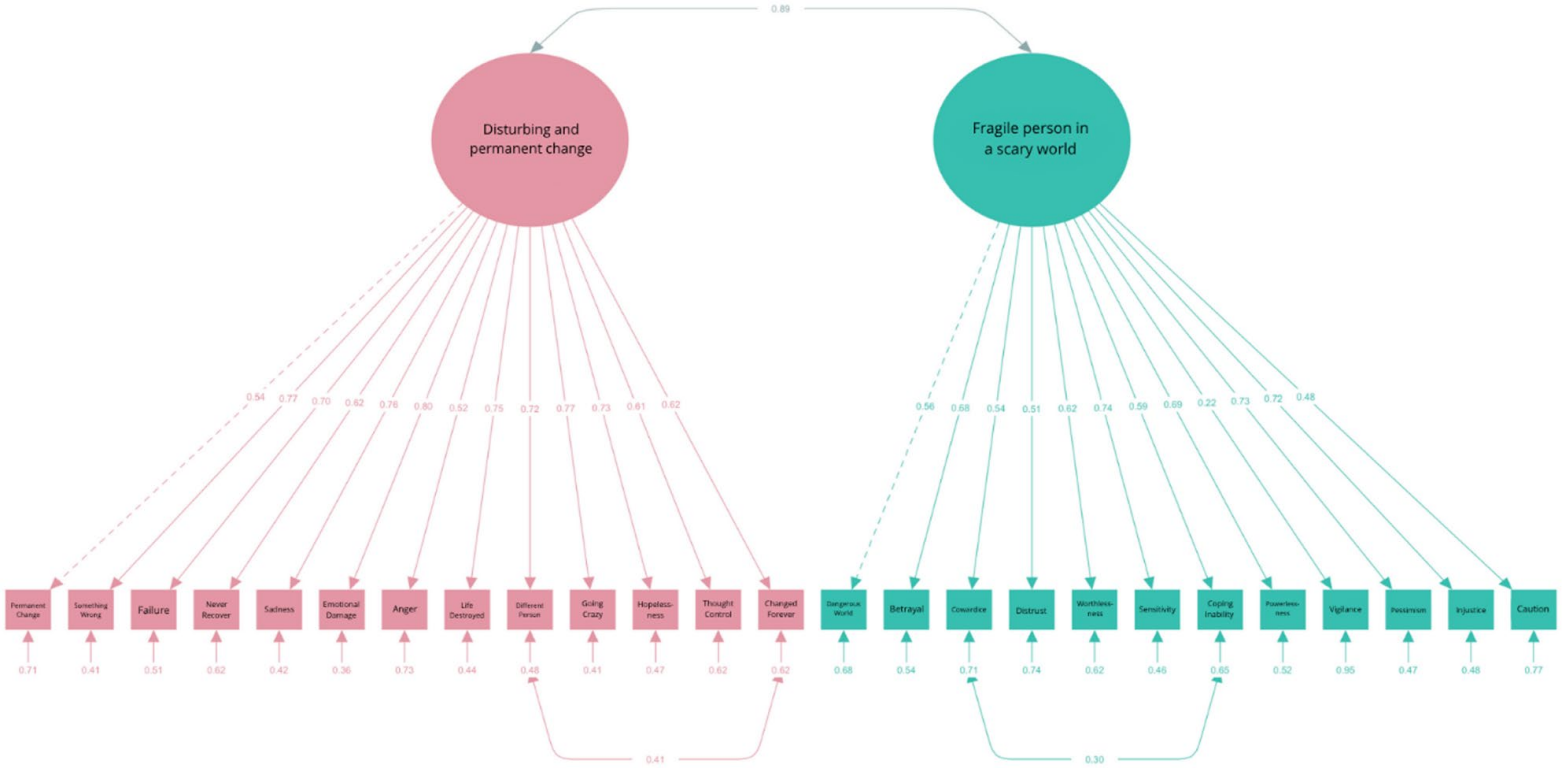
Path Diagram of the Revised Two-Factor Model of the CPTCI

Model refinement was conducted by allowing correlated error terms between item 20 (“I feel like I am a different person since the frightening event.“) and item 24 (“The frightening event has changed me forever.“), and between item 3 (“I am a coward.“) and item 10 (“I can’t cope when things get tough.“), based on modification indices of 48.87 and 26.76, respectively, indicating substantial correlations between these manifest variables (see Table 4; Fig. 4, and Fig. 5). This may be explained by the fact that the items have comparable content but are phrased differently. Since these model revisions do not impact the subscale structure of the CPTCI, we proceeded with our analyses using its original subscale structure.

**Fig. 6:**
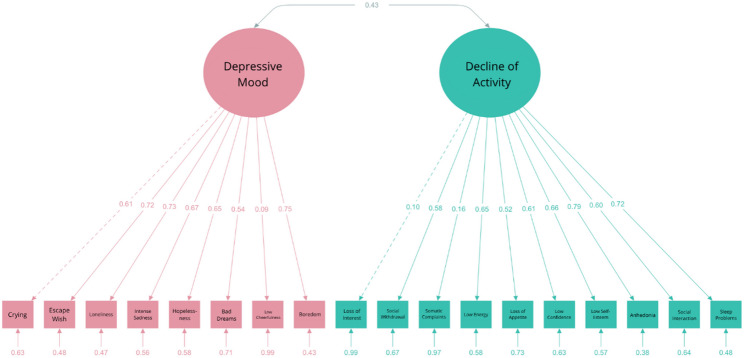
Path Diagram of the Two-Factor Model of the DSRS-C

*DSRS-C.* For the DSRS- C, we tested two models including a mono-factorial structure (22) and a two-factor structure with Decline of Activity and Enjoyment as one factor and Depressive Mood as the second factor [32]. The DSRS-C questionnaire exhibited a non-normal distribution (*W* = 0.956, *p* < .001), prompting the use of the MLR estimation method. Using the Lavaan package, a one-factor model and a two-factor model of the DSRS-C were evaluated. The two-factor model demonstrated a significantly better fit than the one-factor model (see Table 5). Although the primary fit indices (CFI, TLI, SRMR) did not meet the predefined thresholds, the AIC and BIC values favored the two-factor model. Most factor loadings exceeded 0.5, except for item 16 (“I am easily cheered up.“), item 1 (“I look forward to things as much as I used to.”) and Item 6 (“I get tummy aches.“), which had lower factor loadings on their respective latent variables (Fig. 6). The latent variables exhibited a moderate correlation (*r* = .43), supporting their distinction.

**Table 5:** Fit indices of the DSRS-C models

*n* = 208	χ^2^ (df)	SRMR	RMSEA	90% CI	CFI	TLI	AIC	BIC
Cut-off values		≤ 0.08	≤ 0.08	≤ 0.08	≥ 0.90	≥ 0.90		
2-factor model	298.107 (134)	0.111	0.077	0.066, 0.087	0.819	0.793	6049.31	6172.80
1-factor model	671.721 (135)	0.134	0.138	0.143, 0.166	0.573	0.516	6340.19	6460.34
Revised model	199.724 (131)	0.070	0.050	0.037, 0.062	0.924	0.911	5930.80	6064.30

Note. DSRS-C = Depression Self-Rating Scale for Children

**Fig. 7:**
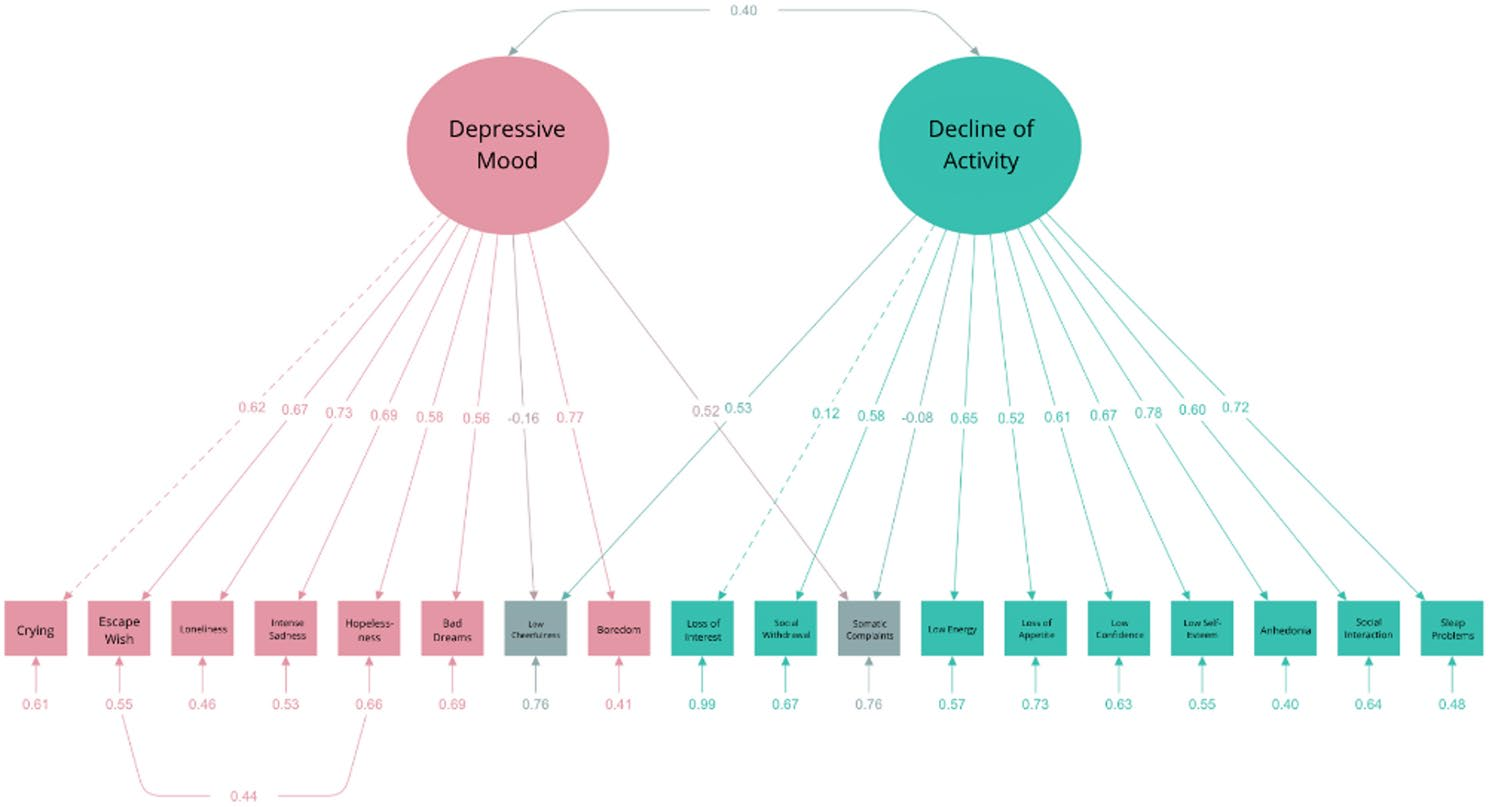
Path Diagram of the Revised Two-Factor Model of the DSRS-C

To address localized areas of model misfit, theoretically justifiable residual covariances were freed between (a) item 6 (“I get tummy aches.“) and the Depressive Mood latent variable (MI = 40.31), consistent with the commonly observed association of psychosomatic symptoms with depressive affect; (b) item 5 (“I feel like running away.“) and item 10 (“I think life isn’t worth living.“) (MI = 40.54), possibly reflecting their conceptual overlap in expressing despair and escape tendencies; and (c) the Decline of Activity and Enjoyment latent variable and item 16 (“I am easily cheered up.”) (MI = 39.12), both pertaining to aspects of positive affect. These adjustments resulted in improved overall fit of the model (see Table 5; Fig. 7). Given that additional empirical support is required before modifying the original single-factor structure of the DSRS-C, subsequent analyses were conducted using the unaltered measurement model.

### Internal consistency

Overall, the CRIES-13, CPTCI, and DSRS-C total and subscales demonstrated satisfactory (Cronbach’s alpha ≥ 0.70) to excellent (Cronbach’s alpha ≥ 0.90) internal consistency, with the exception of the Arousal subscale of the CRIES-13 (Cronbach’s alpha ≥ 0.60) (see Table 6).^1^

**Table 6:** Internal consistencies of the three questionnaires and their subscales

Questionnaire	Overall α	Subscale	Subscale α
		Intrusion	0.835
CRIES–13	0.853	Avoidance	0.755
		Arousal	0.652
CPTCI	0.937	Disturbing and permanent change	0.915
		Fragile person in a scary world	0.863
DSRS–C	0.835	Depressive Mood	0.820
		Decline of Activity and Enjoyment	0.752

Note. CRIES-13 = Children’s Revised Impact of Event Scale; CPTCI = Child Post-Traumatic Cognitions Inventory; DSRS-C = Depression Self-Rating Scale for Children

### Convergent validity

Convergent validity was assessed by examining the associations between the CRIES-13, CPTCI, DSRS-C, and trauma history scores (number of traumatic events), using Pearson’s correlations. As expected, we observed significant moderate positive correlations (0.33 to 0.71) between the questionnaire and trauma history scores, indicating that higher trauma exposure is associated with more trauma-related symptomatology. In addition, CRIES-13, CPTCI, and DSRS-C scores were positively associated with more intense types of exposure as captured by our trauma severity index, and negatively associated with time elapsed since the most recent traumatic event (see Table 7). Finally, compared to non-interpersonal trauma, interpersonal trauma was associated with significantly higher scores on the CRIES-13 (*M* = 19.30 vs. *M* = 12.92, *t*(298.33) = -4.50, *p* < .001), CPTCI (*M* = 46.79 vs. *M* = 38.46, *t*(287.82) = -4.88, *p* < .001), and DSRS-C (*M* = 9.95 vs. *M* = 7.05, *t*(286.41) = -4.08, *p* < .001) scores.

**Table 7:** Pearson’s correlations between CRIES-13, CPTCI, DSRS-C, and trauma history scores

	CRIES–13	CPTCI	DSRS–C
CRIES–13	-		
CPTCI	0.61*	-	
DSRS–C	0.43*	0.71*	-
Trauma History Score	0.35*	0.33*	0.34*
Trauma Severity Index	0.38**	0.36**	0.39**
Time Since Trauma	-0.29**	-0.30**	-0.39**

Note. CRIES-13 = Children’s Revised Impact of Event Scale; CPTCI = Child Post-Traumatic Cognitions Inventory; DSRS-C = Depression Self-Rating Scale for Children. *p < .01; **p < .001

## Discussion

This study evaluated the internal structure and convergent validity of three age- and culture-appropriate questionnaires that assess posttraumatic stress symptoms (CRIES-13), trauma-related cognitions (CPTCI), and comorbid depression symptoms (DSRS-C) in Hindi-speaking children and adolescents in India. The factor structures of all three questionnaires aligned with prior research and our expectations. Specifically, the CRIES-13 exhibited a three-factor structure, while both the CPTCI and the DSRS-C showed a two-factor structure. Additionally, except the Arousal subscale of the CRIES-13, all three questionnaires demonstrated satisfactory internal consistency and convergent validity, which is also consistent with previous research and our hypotheses.

Although our participants were not recruited from clinical populations, a considerable proportion of our sample exhibited scores suggestive of the possible presence of PTSD and Major Depressive Disorder (MDD). To put this into perspective, a previous study estimated the prevalence of PTSD and MDD in Tibetan refugee children who had fled from the military occupation of their country [48]. Their results showed that 11.5% of the children met DSM-IV criteria for both PTSD and MDD, with children who had arrived more recently displaying a higher prevalence of PTSD. It is important to note that our findings are based on self-report questionnaires and do not justify a formal diagnosis of PTSD or MDD. Nevertheless, they underscore the significant burden on Indian children and adolescents in terms of trauma exposure and trauma-related symptoms and highlight the need for further research and effective interventions to support their mental health and overall well-being.

The guiding purpose of our research into culturally adapting as well as providing a preliminary psychometric evaluation of these questionnaires is to serve clinical utility [49] as well as clinical research in India. Clinically, these validated tools may serve inclusive screening and diagnosis by reducing language and cultural barriers to care and by providing a standardized impression of the severity of different types of trauma-related complaints, which in turn may promote efficient use of scarce mental health care resources. Research into (for example) the prevalence of psychotrauma in India may be amplified by the introduction of these internationally standardized measures, making benchmarking and subgroup analyses more feasible.

Several limitations of our study should be acknowledged. First, our participants completed the questionnaires without supervision (unproctored administration). Future studies should therefore consider data collection under adult supervision. Relatedly, although a comprehension check suggested a good understanding of the questionnaires, it is important to note the absence of a benchmark for this. Second, our data did not allow for testing of additional important psychometric properties such as the diagnostic accuracy, test-retest reliability, discriminant validity, and measurement invariance of our questionnaires, which therefore need to be ascertained in future research. Future research could also focus on adapting and validating additional trauma-related instruments relevant to clinical practice and research in India, such as the 10-item Children’s Post-Traumatic Cognitions Inventory-Short Form (CPTCI-S) [50]. Third, although the sample size was adequate for our analyses, we acknowledge that its convenience nature and the relatively low completion rate pose limitations for generalizabilty. For example, symptomatic children may have been more likely to avoid participation compared to non-symptomatic participants. Furthermore, despite the steady rise in internet penetration and smartphone usage in India [51], restricted access to these technologies may have contributed to additional selection bias. Fourth, while Hindi is the most widely spoken official language in India, it is mostly spoken in northern and central India. Given the multitude of languages and dialects spoken in India (estimated at 122 major languages and 1599 other languages) [52], it is important to adapt and validate the questionnaires for use in other Indian languages. This is especially relevant in rural areas where English or Hindi may not be commonly spoken.

Despite these limitations and the need for further evaluation, our findings suggest that the current Hindi versions of the questionnaires may serve as low-cost tools for assessing posttraumatic stress symptoms (CRIES-13), trauma-related cognitions (CPTCI), and depressive symptoms (DSRS-C) in children and adolescents in India. Their brevity and self-report format enhance ease of administration and may support efforts to address the high prevalence of these symptoms among Indian youth. Our findings—that previously observed factor structures of these questionnaires, as well as their associations with trauma exposure, appear to be replicated in the present sample—provide initial support for their validity in an Indian context. However, consistent with the DSM-5, we emphasize that the validity of existing instruments across cultures should always be carefully considered and never assumed, given cultural influences that shape trauma-related and depressive symptoms [2].

## Data Availability

The datasets used and/or analysed during the current study are available from the corresponding author on reasonable request.

## Appendix A: **Children’s Revised Impact of Event Scale (CRIES-13)**^2^

### Instructions

नीचे कुछ बयानों की सूची दी गई है जो लोग तनावपूर्ण जीवन घटनाओं के बाद अनुभव कर सकते हैं। कृपया हर बयान के लिए यह बताएं कि पिछले सात दिनों में वह आपके लिए कितनी बार सही रहा। यदि वह अनुभव पिछले सात दिनों में बिल्कुल नहीं हुआ, तो ‘बिलकुल नहीं’ वाले बॉक्स को चिह्नित करें।.

*Below is a list of statements describing things people may experience after stressful life events. Please indicate how often each statement was true for you during the past seven days. If you did not have that experience during this time*,* please tick the ‘Not at all’ box.*

Below is a list of comments made by people after a stressful life event. Please tick each item showing how frequently these comments were true for you during the past seven days. If they did not occur during that time please tick the ‘not at all’ box.

1. क्या आप न चाहते हुए भी उस घटना के बारे में सोचते हैं?

*Do you think about that incident even if you don’t want to?*

Do you think about it even when you don’t mean to?

2. क्या आप उसे अपने दिमाग से निकालना चाहते हैं?

*Do you try to remove it from your memory?*

Do you try to remove it from your memory?

3. क्या आपको ध्यान देने में मुश्किल होती है?

*Do you have difficulty paying attention?*

Do you have difficulties paying attention or concentrating?

4. क्या आप उस घटना के बारे में सोचने से भावुक हो जाते हैं?

*Do you have waves of strong feelings about it?*

Do you have waves of strong feelings about it?

5. क्या आप उस घटना के बाद आसानी से चौंकते या घबरा जाते हैं?

*Do you startle more easily or feel more nervous than you did before?*

Do you startle more easily or feel more nervous than you did before it happened?

6. क्या आप उस घटना की याद दिलाने वाली जगहों और परिस्थितियों से दूर रहते हैं?

*Do you avoid places and situations that remind you of the incident?*

Do you stay away from reminders of it (e.g. places or situations)?

7. क्या आप कोशिश करते हैं उस घटना के बारे में न बात करने की?

*Do you try not to talk about the incident?*

Do you try not talk about it?

8. क्या उस घटना की तस्वीरें आपके दिमाग में अचानक आ जाती हैं?

*Do images of the incident suddenly flash into your mind?*

Do pictures about it pop into your mind?

9. क्या दूसरी चीज़ें आपको उस घटना की याद दिलाती हैं?

*Do other things suddenly remind you of the incident?*

Do other things keep making you think about it?

10. क्या आप कोशिश करते हैं उस घटना के बारे में नहीं सोचने की?

*Do you try not to think about the incident?*

Do you try not to think about it?

11. क्या आप आसानी से चिड़ जाते हैं?

*Do you get irritated easily?*

Do you get easily irritable?

12. जरूरत न होने पर भी क्या आप सतर्क और सहज रहते हैं?

*Are you alert and watchful even when there is no need to be?*

Are you alert and watchful even when there is no obvious need to be?

13. क्या आपको सोने में समस्या होती है?

*Do you have trouble sleeping?*

Do you have sleep problems?

### Scoring instructions

Each item is rated on a 4-point Likert scale:

0 = बिल्कुल नहीं (Not at all).

1 = कभी कभी (Rarely).

3 = कई बार (Sometimes).

5 = बार बार (Often).

There are no reverse-scored items. The total score is the sum of all item scores (range: 0 to 65). Subscales scores are computed as follows:

Intrusion: Items 1, 4, 8, 9.

Avoidance: Items 2, 6, 7, 10.

Arousal: Items 3, 5, 11, 12, 13.

Each subscale score is the sum of its item scores. Higher scores indicate greater posttraumatic stress symptoms, with a total score of 30 or above suggesting clinically relevant symptom levels.

## Appendix B: Child Post-Traumatic Cognitions Inventory (CPTCI)^3^

### Instructions

हम यह जानना चाहते हैं कि उस डरावनी घटना के बाद आपके मन में किस तरह के विचार और भावनाएँ आ रही हैं। नीचे कुछ बयानों की सूची दी गई है। कृपया प्रत्येक बयान को ध्यान से पढ़ें और यह बताएं कि आप उससे कितनी सहमत या असहमत हैं, इसके लिए उपयुक्त बॉक्स पर निशान लगाएँ। लोग डरावनी घटनाओं पर अलग-अलग तरीकों से प्रतिक्रिया करते हैं। इन बयानों का कोई सही या गलत उत्तर नहीं है।.

*We would like to know what kinds of thoughts and feelings you’ve been having after the frightening event. Below is a list of statements. Please read each one carefully and indicate how much you agree or disagree with each statement by ticking the appropriate box. People respond to frightening events in different ways. There are no right or wrong answers to these statements.*

We would like to know what kinds of thoughts and feelings you’ve been having after the frightening event. Below is a list of statements. Please read each statement carefully and tell us how much you AGREE or DISAGREE with each statement by ticking one box. People react to frightening events in many different ways. There are no right or wrong answers to these statements.

1. मुझे कोई भी चोट पहुंचा सकता है।.

*Anyone could hurt me.*

Anyone could hurt me.

2. हर कोई मुझे निराश करता है।.

*Everyone lets me down.*

Everyone lets me down.

3. मैं डरपोक हूँ।.

*I am a coward.*

I am a coward.

4. उस घटना के बाद से मेरी प्रतिक्रियाओं का मतलब है कि मैं बदतर के लिए बदल गया हूं।.

*My reactions since the frightening event mean I have changed for the worse.*

My reactions since the frightening event mean I have changed for the worse.

5. मुझे लोगों पर भरोसा नहीं है।.

*I don’t trust people.*

I don’t trust people.

6. उस घटना के बाद से मेरी प्रतिक्रियाओं का मतलब है कि मेरे साथ कुछ गंभीर रूप से गलत है।.

*My reactions since that incident mean there is something seriously wrong with me.*

My reactions since the frightening event mean something is seriously wrong with me.

7. मुझमें कोई अच्छाई नहीं है।.

*I have no goodness in me.*

I am no good.

8. अपने डर-भय को न भूल पाने का मतलब है की मैं असफल हूँ।.

*Not being able to let go of my fears means I am a failure.*

Not being able to get over all my fears means that I am a failure.

9. छोटी छोटी बातें मुझे परेशान करती हैं।.

*Small things upset me.*

Small things upset me.

10. मैं कठिनाइयों का सामना नहीं कर पाता/पाती हूँ।.

*I am not able to cope when things get tough.*

I can’t cope when things get tough.

11. मैं अपने साथ बुरी चीज़ें होने से नहीं रोक सकता/सकती हूँ।.

*I can’t stop bad things from happening to me.*

I can’t stop bad things from happening to me.

12. मुझे हमेशा खतरों से सतर्क रहना है।.

*I have to watch out for dangers all the time.*

I have to watch out for danger all the time.

13. उस घटना के बाद मेरी प्रतिक्रियाओं से लगता है की मैं उसे कभी भूल नहीं पाउँगा।.

*My reactions since the frightening event mean I will never get over it.*

My reactions since the frightening event mean I will never get over it.

14. मैं पहले खुश रहता/रहती थी, पर उस घटना के बाद हमेशा दुखी रहता/रहती हूँ।.

*I used to be a happy person*,* but after that incident I am always sad.*

I used to be a happy person but now I am always sad.

15. हमेशा बुरा होता है।.

*Bad things always happen.*

Bad things always happen.

16. मैं फिर कभी सामान्य मनोभाव नहीं रख पाउँगा।.

*I will never be able to have normal feelings again.*

I will never be able to have normal feelings again.

17. मुझे डर लगता है कि मैं गुस्से में कुछ तोड़-फोड़ न कर दूँ या किसी को घायल न कर दूँ।.

*I’m scared that I’ll get so angry that I’ll break something or hurt someone.*

I’m scared that I’ll get so angry that I’ll break something or hurt someone.

18. ज़िंदगी अच्छी नहीं है।.

*Life is not fair.*

Life is not fair.

19. उस घटना से मेरा जीवन बर्बाद हो गया है।.

*That incident ruined my life.*

My life has been destroyed by the frightening event.

20. मुझे लगता है कि उस डरावनी घटना के बाद मैं एक अलग व्यक्ति हूँ।.

*I feel like I am a different person since the frightening event.*

I feel like I am a different person since the frightening event.

21. उस घटना के बाद मेरी प्रतिक्रियाओं से ऐसा लगता है कि मैं पागल हो जाऊंगा।.

*My reactions since the frightening event show that I must be going crazy.*

My reactions since the frightening event show that I must be going crazy.

22. अब मेरे साथ कुछ भी अच्छा नहीं हो सकता।.

*Nothing good can happen to me anymore.*

Nothing good can happen to me anymore.

23. अगर मैं उस घटना की सोच का नियंत्रण न करूँ, तो मेरे साथ कुछ भयंकर होगा।.

*Something terrible will happen if I do not try to control my thoughts about the frightening event.*

Something terrible will happen if I do not try to control my thoughts about the frightening event.

24. उस डरावनी घटना ने मुझे हमेशा के लिए बदल दिया है।.

*The frightening event has changed me forever.*

The frightening event has changed me forever.

25. मुझे बहुत सावधान रहना है क्योंकि मेरे साथ कुछ बुरा हो सकता है।.

*I have to be really careful because something bad could happen.*

I have to be really careful because something bad could happen.

### Scoring instructions

Each item is rated on a 4-point Likert scale:

1 = पूर्ण असहमति (Strongly disagree).

2 = थोड़ी असहमति (Slightly disagree).

3 = थोड़ी सहमति (Slightly agree).

4 = पूर्ण सहमति (Strongly agree).

There are no reverse-scored items. The total score is the sum of all item scores (range: 25 to 100). Subscale scores are computed as follows:

Permanent and Disturbing Change (PC): Items: 4, 6, 8, 13, 14, 16, 17, 19, 20, 21, 22, 23, 24.

Fragile Person in a Scary World (SW): Items: 1, 2, 3, 5, 7, 9, 10, 11, 12, 15, 18, 25.

Each subscale score is the sum of its item scores. Higher scores indicate more negative trauma-related cognitions. A total score of 46 to 48 or higher is considered clinically significant, indicating trauma-related cognitions typical of children and adolescents with PTSD.

## Appendix C: Depression Self-Rating Scale for Children (DSRS-C)^4^

### Instructions

कृपया नीचे दिए गए वाक्यों को ध्यान से पढ़िए और पिछले सप्ताह आपने कैसा अनुभव किया, उसका उत्तर दीजिए। यह महत्वपूर्ण है कि आप ईमानदारी से जवाब दें। सही उत्तर वही है जो यह बताता है कि आपने वास्तव में कैसा महसूस किया है।.

*Please read the statements below carefully and answer based on how you have felt during the past week. It is important that you respond honestly. The correct answer is the one that reflects how you have truly felt.*

Please read these statements and tick the answer that best describes how you have felt during the past week. It is important to answer as honestly as you can. The correct answer is to say how you have really felt.

1. मैं पहले की तरह चीज़ों की प्रतीक्षा करता/करती हूँ।.

*I look forward to things as much as I used to.*

I look forward to things as much as I used to.

2. मैं अच्छे से सोता/सोती हूँ।.

*I sleep very well.*

I sleep very well.

3. मुझे रोने का मन करता है।.

*I feel like crying.*

I feel like crying.

4. मैं बाहर खेलने जाना पसंद करता/करती हूँ।.

*I like to go out to play.*

I like to go out to play.

5. मुझे भाग जाने का मन करता है।.

*I feel like running away.*

I feel like running away.

6. मेरा पेट दर्द करता है।.

*I get tummy aches.*

I get tummy aches.

7. मुझमें बहुत शक्ति है।.

*I have lots of energy.*

I have lots of energy.

8. मुझे खाना खाना पसंद है।.

*I enjoy my food.*

I enjoy my food.

9. मैं अपने लिए खड़ा हो सकता/सकती हूँ।.

*I can stand up for myself.*

I can stick up for myself.

10. मुझे लगता है की ज़िन्दगी जीने के लायक नहीं है।.

*I think life isn’t worth living.*

I think life isn’t worth living.

11. मैं जो भी करता/करती हूँ उसमें अच्छा/अच्छी हूँ।.

*I am good at the things I do.*

I am good at the things I do.

12. मैं सभी चीजों का पहले की तरह ही आनंद लेता/लेती हूँ।.

*I enjoy the things I do as much as I used to.*

I enjoy the things I do as much as I used to.

13. मुझे अपने परिवार से बात करना पसंद है।.

*I like talking with my family.*

I like talking with my family.

14. मुझे बुरे सपने आते हैं।.

*I have bad dreams.*

I have bad dreams.

15. मैं बहुत अकेलापन महसूस करता/करती हूँ।.

*I feel very lonely.*

I feel very lonely.

16. मैं आसानी से खुश हो जाता/जाती हूँ।.

*I am easily cheered up.*

I am easily cheered up.

17. मुझे बहुत दुःख होता है और मैं उसे नहीं सह पाता/पाती हूँ।.

*I feel so sad I can hardly stand it.*

I feel so sad I can hardly stand it.

18. मैं बहुत नीरस रहता/रहती हूँ।.

I feel very bored.

I feel very bored.

### Scoring instructions

Each item is rated on a 3-point Likert scale:

काफी हदतक (Mostly) = 2 or 0 (depending on the item direction).

कभी कभी (Sometimes) = 1.

कभी नही (Never) = 0 or 2 (depending on the item direction).

Items are reverse-scored as follows:

Positively worded items [1, 2, 4, 7–9, 11–13, 16]: Mostly = 0, Sometimes = 1, Never = 2.

Negatively worded items [3, 5, 6, 10, 14, 15, 17, 18]: Mostly = 2, Sometimes = 1, Never = 0.

The total score is the sum of all item scores (range: 0 to 36). Higher scores indicate more severe depressive symptoms.

## Abbreviations

CRIES-13: Children’s Revised Impact of Event Scale
CPTCI: Child Post-Traumatic Cognitions Inventory
DSRS-C: Depression Self-Rating Scale for Children
PTSD: Posttraumatic Stress Disorder
CFA: Confirmatory Factor Analysis
MLR: Robust Maximum Likelihood
SRMR: Standardized Root Mean Square Residual
RMSEA: Root Mean Square Error of Approximation
CFI: Comparative Fit Index
TLI: Tucker-Lewis Index
AIC: Akaike Information Criterion
BIC: Bayesian Information Criterion
MI: Modification Indices
MDD: Major Depressive Disorder

## Footnotes

1 Sensitivity analyses for (≤ 12 years) versus older (≥ 13) children showed that internal consistencies for both age groups were good to excellent across all scales (data available from the authors).
